# Self-assembly preparation of SiO_2_@Ni-Al layered double hydroxide composites and their enhanced electrorheological characteristics

**DOI:** 10.1038/srep18367

**Published:** 2015-12-16

**Authors:** Xuqiang Ji, Wenling Zhang, Lei Shan, Yu Tian, Jingquan Liu

**Affiliations:** 1 College of Chemical Science and Engineering, Laboratory of Fiber Materials and Modern Textile, The Growing Base for State Key Laboratory, Collaborative Innovation Centre for Marine Biomass Fibers, Qingdao University, Qingdao 266071, China; 2 State Key Laboratory of Tribology, Tsinghua University, Beijing 100084, China

## Abstract

The core-shell structured SiO_2_@Ni-Al layered double hydroxide (LDH) composites were prepared via self-assembly of Ni-Al LDH on the surface of SiO_2_ spheres. Only coating a layer of ultrathin Ni-Al LDH sheet, the resulting SiO_2_@Ni-Al LDH composites exhibit significantly enhanced electrorheological (ER) characteristics compared to conventional bare SiO_2_ spheres. The monodispersed SiO_2_ spheres with average diameters of 260 nm were synthesized by the hydrolysis of tetraethyl orthosilicate (TEOS), while the shell part, Ni-Al LDH sheet was prepared by the hydrothermal procedure. The morphology of the samples was investigated via scanning transmission electron microscopy (STEM), scanning electron microscopy (SEM) and transmission electron microscopy (TEM). The structure of the samples was characterized by X-ray diffraction (XRD). The species and distribution of elements in samples were confirmed by X-ray photoelectron spectroscopy (XPS), Energy dispersive analysis of X-ray (EDX) and elemental mapping in STEM. Subsequently, the ER characteristics of the composites dispersed in insulating oil were characterized by a rotational rheometer. The electric field-stimulated rheological performances (yield stress, viscosity, modulus, etc.) were observed under an external electric field, which is different from the Newtonian state in the free electric field.

There are many examples of biological and inorganic materials with complicated and efficient hierarchical morphologies in natural world, such as marine coccolith, radiolarian shell and clay, exhibiting superior performance^1^,^2^,^3^,^4^,^5^. To synthesize inorganic materials with natural materials’ structure for the development of catalysis^6^, electronic^7^, lithium secondary battery^8^, and many other applications to meet the challenges of green energy and sustainability, has been one of the hotspots in material chemistry fields. Here, the layered double hydroxides (LDH) are hydrotalcite-like clays with the empirical formula, [M^II^_1-x_M^III^_x_(OH)_2_]^x+^[A^n−^]_x/n_·mH_2_O, where M^II^ and M^III^ are divalent and trivalent metals such as Mg, Ni, Co, Al , and Fe, A^n−^ can be almost any anion with the charge number of n^9^,^10^,^11^. Due to their special and complicated structure and anion exchange property, LDH materials have been applied to many areas including catalysts^12^,^13^,^14^,^15^,^16^, anion exchangers^17^,^18^,^19^,^20^, electrodes for alkaline secondary batteries and supercapacitor^21^,^22^, especially for electrorheological (ER) fluids^23^,^24^,^25^. Compared to other clay minerals including smectites, kaolins, Pyrophyllite, talo etc., LDH sheets possess distinctive advantages, such as positive charges on its surface due to the existence of metal ions (Ni^+^, Al^3+^, Mg^2+^ etc.) and negative charges among the interlayers^26^, which are more sensitive to electric stimulus for the application of ER systems. ER fluids are smart materials that experience continuous and reverse changes in rheological properties in a few milliseconds under an applied electric field^27^,^28^,^29^,^30^. In general, it involves a dispersed phase such as polyaniline (PANI), polypyrrole (PPy), polystyrene (PS), silica, titania, zeolite, clay or other polarizable or semiconducting particles and a continuous phase, including silicone oil or corn oil, etc.^24^,^31^,^32^,^33^. The solid-like property emerges when electric field is applied across the ER fluids, with the formation of chain-like or columnar structures aligned to the electric field. Due to the fast response to electric field, controllable mechanical properties and no waste discharge, the ER fluid has been paid more and more attention in an automotive industry such as shock absorbers, engine mounts and clutches^34^,^35^.

The inorganic materials (aluminosilicate, zeolite, alumina, TiO_2_, SiO_2_ etc.) and the semiconducting polymer (PANI, copolypyrrole, copolyaniline, polyacene quinone radicals etc.) have been widely utilized to prepare ER suspensions due to their low cost and anisotropic morphology^36^,^37^,^38^,^39^,^40^,^41^,^42^,^43^,^44^,^45^,^46^,^47^,^48^. Recently, much attention has been focused on the layered inorganic materials, such as LDH sheet, graphene or graphene oxide (GO) based hybrids. However, the aggregation of LDH sheet usually greatly weakens the material’s ER properties, thus significantly limits the applications in an automotive industry field. The core-shell structured composites with controllable morphology, orientation, and dimensionality have evoked considerable interest because of their remarkable ER properties, low cost and well controlled particle size^49^,^50^.

Diverse composite nanoparticles with core-shell structure have been found to show excellent ER properties. PS microspheres coated with GO sheets were prepared using π–π stacking interactions^51^. PANI-coated anisotropic snowman-like poly(methyl methacrylate) (PMMA) particles were also prepared with an anionic surfactant as ER fluid suspension particles^52^. Core-shell structured titania/PANI and PS/PANI microspherical composites were synthesized via *in situ* polymerization in the presence of titanium oxide or PS nanospheres^42^,^53^. SiO_2_/TiO_2_ particles were prepared by means of a co-precipitation process by Wu and his coworkers^54^. Monodispersed PMMA microbeads coated with MWNT–NH_2_ were also prepared via a grafting reaction^55^. To prepare core-shell structured biomimetic materials, Mirkin and co-workers have explored diatom silica walls as the core template to integrate with inorganic nanoparticles. The nanoparticles formed a near-monolayer along the surface morphology and shape of the diatom template^56^. And a facile approach related to direct self-assembly of inorganic nanostructures under mild conditions without the utilization of harsh chemical treatments is under explored urgently. Seeking for greener synthetic routes to avoid the use of toxic reagents and realize time-saving has been one of the major concerns of the global vision of world economy.

Inspired by the previous work, here, SiO_2_@Ni-Al LDH core-shell structure was prepared via self-assembly of Ni-Al LDH on SiO_2_ sphere as shown in Fig. 1. As the core material, monodispersed SiO_2_ with diameters of 260 nm were synthesized by the hydrolysis of tetraethyl orthosilicate (TEOS) induced using the catalysis of ammonia in ethanol medium, while the shell part, Ni-Al LDH, was prepared by the hydrothermal method. Subsequently, the ER characteristic of the SiO_2_@Ni-Al LDH composite dispersed in insulating oil was characterized by a rotational rheometer.

## Results and Discussion

**Figure 2:**
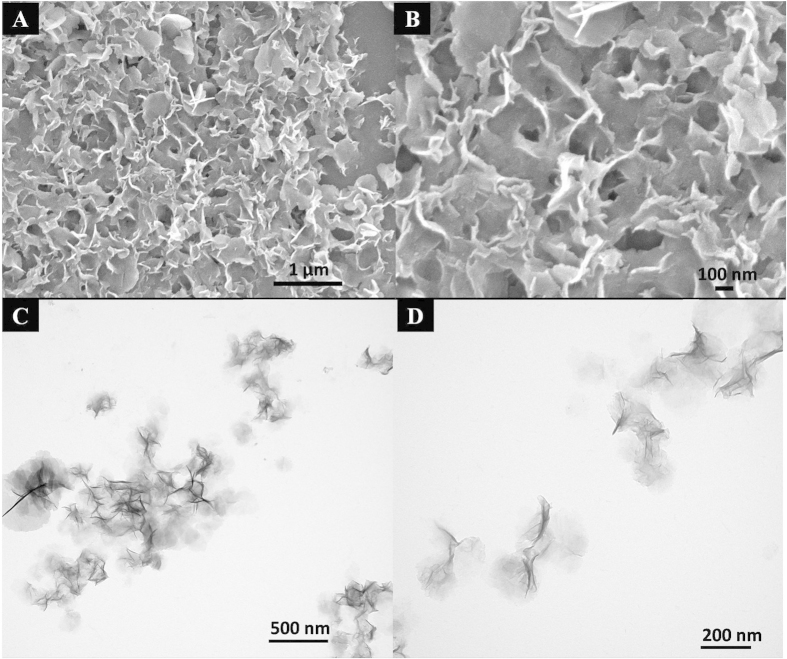
(**A,C**) SEM and TEM images of Ni-Al LDH sheets; (**B,D**) High magnification SEM and TEM images of Ni-Al LDH sheets.

Generally, the surface of the bare SiO_2_ sphere in ethanol is negatively charged with a zeta potential value of −15.8 mV, the zeta potential value for Ni-Al LDH sheets is 16.18 mV. Therefore, the electrostatic interactions between the negatively charged SiO_2_ sphere and positively charged Ni-Al LDH sheet could take place. Due to the low zeta potential, the anionic surfactant was also introduced to enhance the adsorption between SiO_2_ sphere and Ni-Al LDH sheet. The morphology of Ni-Al LDH sheet is shown in Fig. 2. It can be observed from the SEM and TEM images, Ni-Al LDH sheet is irregular and thin hexagonal platelet with a mean lateral size of 300 nm, while most of the LDH sheets stack with each other.

**Figure 3:**
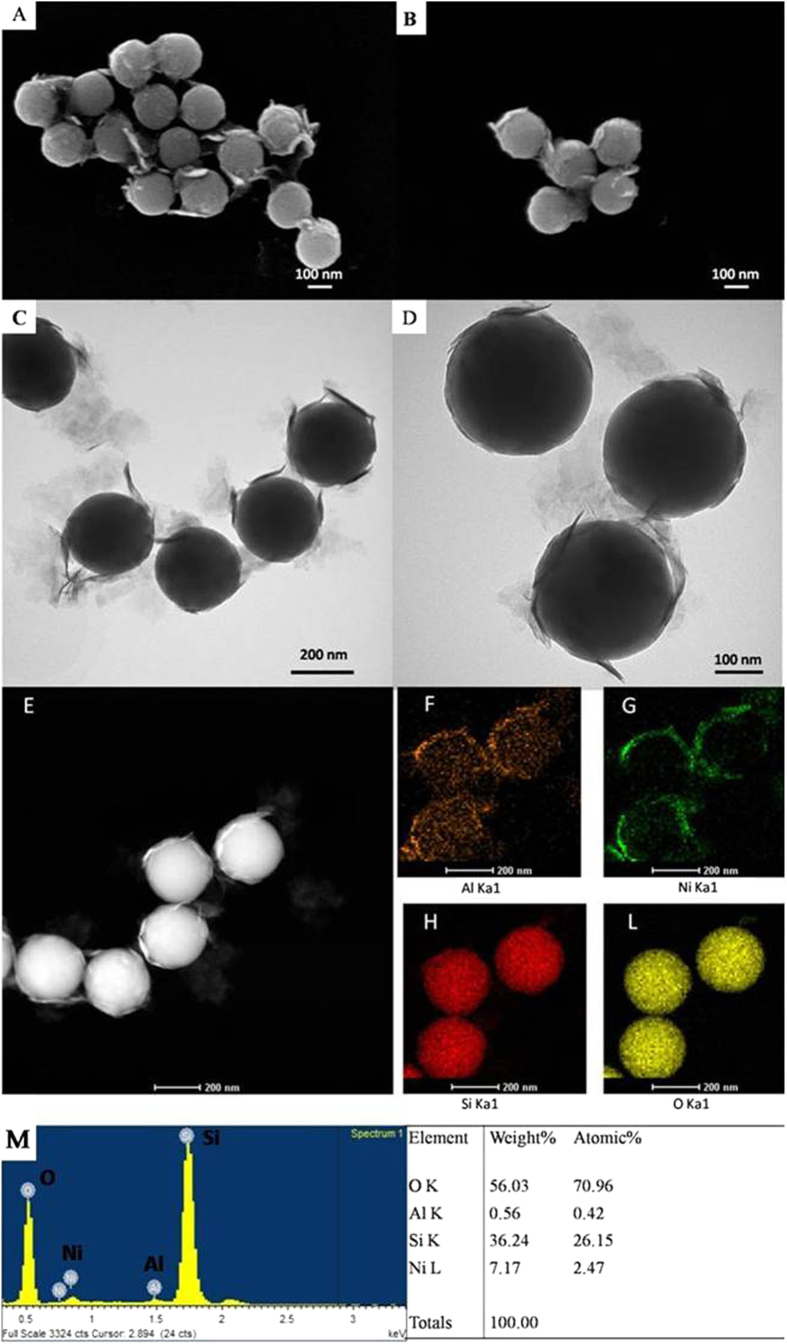
(**A,B**) SEM images (**C,D**) TEM images of SiO_2_@Ni-Al LDH composites; (**E**) Dark-field STEM image of SiO_2_@Ni-Al LDH composites. The elemental mapping for Al (**F**), Ni (**G**), Si (**H**) and O (**L**) elements in the core-shell structure of SiO_2_@Ni-Al LDH composites; and (**M**) EDX spectrum of SiO_2_@Ni-Al LDH composites.

The morphology of SiO_2_@Ni-Al LDH composites was analyzed through SEM (Fig. 3A,B) and TEM (Fig. 3C,D) images. The SiO_2_ spheres are found to be monodispersed with a uniform size of ~260 nm. The final SiO_2_@Ni-Al LDH composites exhibited a roughly spherical morphology with curly Ni-Al LDH sheets coated on the surface. The presence of Ni-Al LDH sheet in the composites was also proved through EDX (Fig. 3M), the weight percentages of Ni and Al elements were calculated to be 7.17% and 0.56% respectively.

A dark-field STEM analysis of SiO_2_@Ni-Al LDH composites was also used to characterize the distribution of the elements and the result is shown in Fig. 3E. It can be seen that the SiO_2_@Ni-Al LDH composites have a representative core-shell structure. In addition, element mapping of the identical spheres showed the space distribution of Si, O, Ni, Al. As shown in Fig. 3H,L, the core part is filled with intense Si and O signals. Meanwhile, the Ni, Al elements are mainly located on the shell part, indicating the successful coating of the Ni-Al LDH sheet.

**Figure 4:**
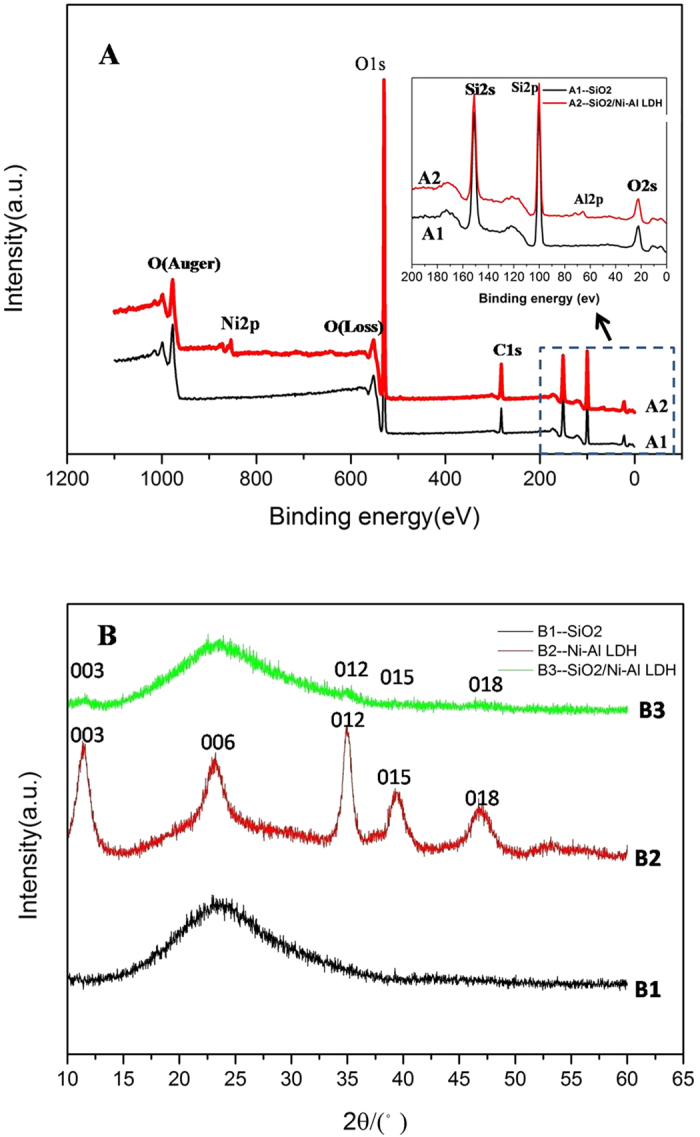
(**A**) XPS survey spectra of SiO_2_ sphere and SiO_2_@Ni–Al LDH composites. (**B**) XRD patterns of SiO_2_ sphere, Ni−Al LDH sheet, and SiO_2_@Ni-Al LDH composites, respectively.

The XRD patterns of SiO_2_ sphere, Ni-Al LDH sheet, and SiO_2_@Ni-Al LDH composites are shown in Fig. 4B. The diffraction pattern of SiO_2_ sphere depicts a reflection characteristic of amorphous silica^57^. After the coating with the Ni-Al LDH sheet, peaks related to 003, 006, 012 and 015 originating from Ni-Al LDH sheet can be observed in addition to those resulted from silica. The composition of SiO_2_ sphere and SiO_2_@Ni-Al LDH composites was investigated using the XPS technique. As shown in Fig. 4A, the XPS survey spectrum of SiO_2_@Ni-Al LDH composites revealed the presence of Si, O, Ni, Al elements, implying the formation of SiO_2_@Ni-Al LDH composites. Taken together, the results of SEM, TEM, EDX, STEM, XRD and XPS evidenced the successful synthesis of SiO_2_@Ni-Al LDH composites.

**Figure 5:**
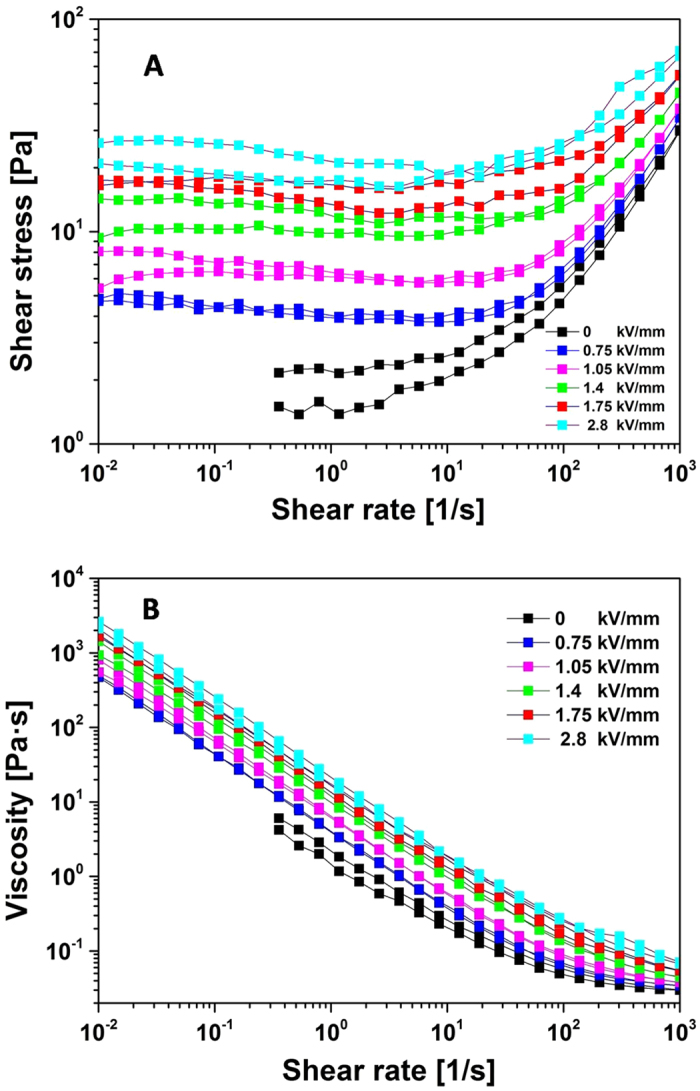
Flow curves of shear stress (**A**) and viscosity (**B**) *vs*. shear rate for the SiO_2_@Ni-Al LDH composites (particle concentration, 10 wt%)-based ER fluid.

**Figure 6:**
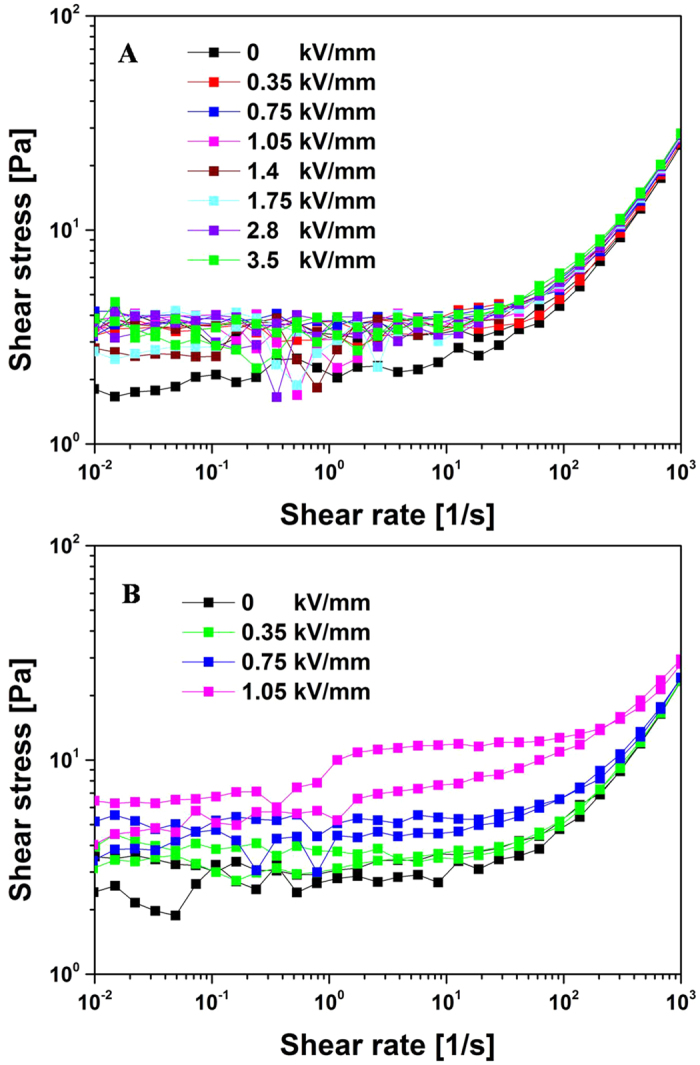
Flow curves of shear stress *vs*. shear rate for the single SiO_2_ particle (**A**) and Ni−Al LDH (**B**) (concentration, 10 wt%)-based ER fluid.

In general, the shear stress of the bare silica particles based suspensions are not very sensitive to the external electric field stimulus due to their weak ER activity^58^. Therefore, coating a layer of ultrathin Ni-Al LDH sheet, is an effective way to enhance the ER activity of the bare SiO_2_ spheres. Here, the prepared SiO_2_@Ni-Al LDH composites exhibited improved ER performance compared to the conventional bare SiO_2_ particles, which have been described below:

The shear stress-shear rate behaviour of the SiO_2_@Ni-Al LDH composites (10 wt% particle concentration) was tested using a rotational rheometer equipped with a high voltage generator subjected to different electric field strengths. As shown in Fig. 5A, the shear stress increased linearly with the increasing shear rate without an external electric field, which is consistent with a Newtonian fluid behaviour^59^. However, when a high electric field was applied, the ER fluid exhibited a large increase in shear stress because of the formation of chain-like structure. This performance was similar to a traditional Bingham fluid. The shear stress of bare SiO_2_ particle based ER fluid exhibited slightly changes with the increasing of electric field strength shown in Fig. 6A, however, pure Ni-Al LDH based ER fluid resulted in electric short at even 1.05 kV/mm due to its relatively higher electric conductivity (Fig. 6B). Figure 5B shows the shear viscosity of SiO_2_@Ni-Al LDH composites as a function of the shear rate. The ER fluid of SiO_2_@Ni-Al LDH composites showed Newtonian fluid characteristics without an electric field and typical shear thinning behaviour^60^ under different electric fields.

**Figure 7:**
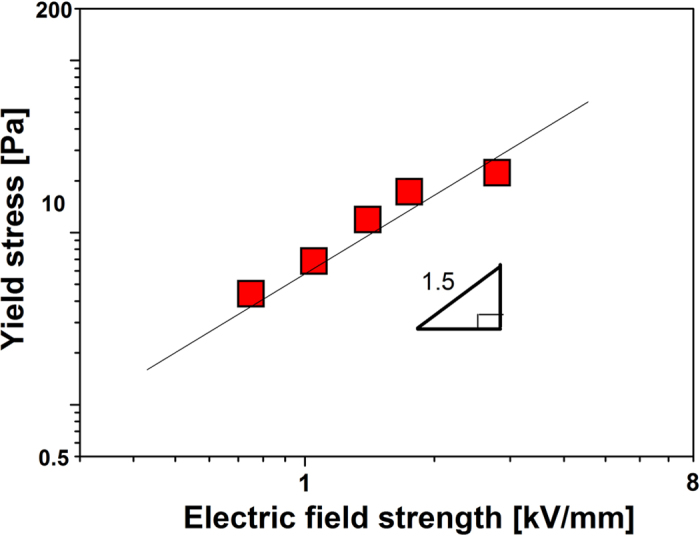
Yield stress as a function of electric field strength for 10 wt % SiO_2_@Ni-Al LDH composite based ER fluid.

Furthermore, the dynamic yield stress was plotted as a function of electric fields in log-log scale shown in Fig. 7. The correlation between the yield stress (τ_y_) and electric fields (E) is fitted by the equation τ_y_ ∝ E^m^, where m equals to 1.5, which is consistent with the conduction model. The conductivity mismatch between the particles and the liquid medium is considered to be the critical factor to result in the aligned array along the field direction in ER system.

**Figure 8:**
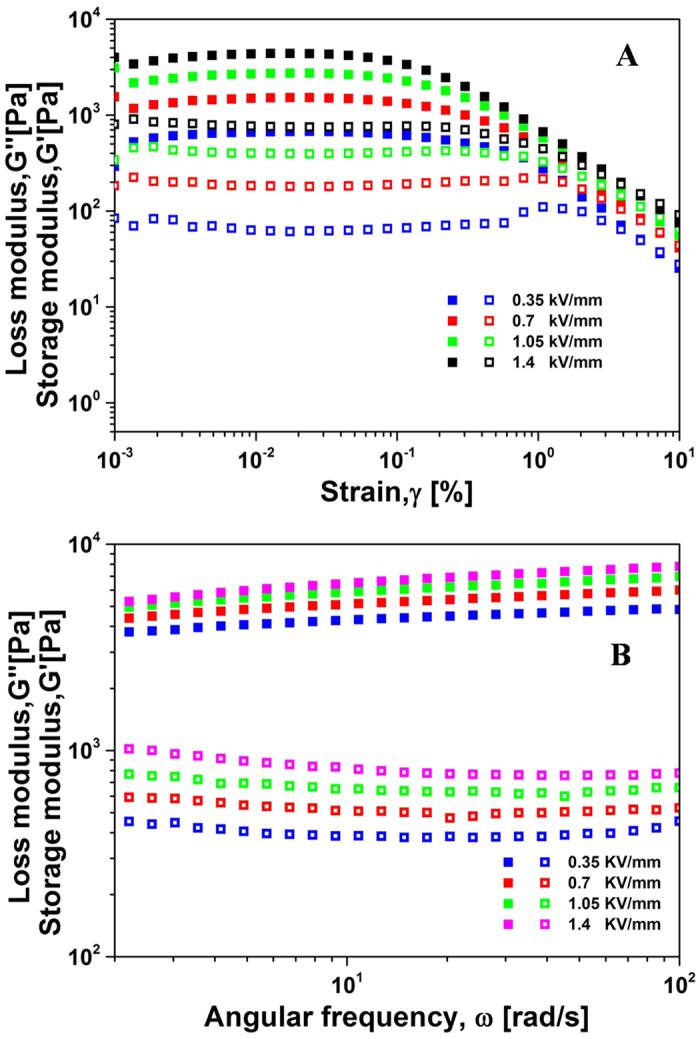
(**A**) Amplitude sweep for SiO_2_@Ni-Al LDH composites (particle concentration, 10 wt%)-based ER fluid at 6.28 rad s^−1^ of angular frequency. (**B**) Frequency sweep for the SiO_2_@Ni-Al LDH composites (particle concentration, 10 wt%)-based ER fluid with a strain amplitude of 0.01% (G′: closed symbols; G″: open symbols).

The viscoelastic properties of the ER fluids was examined by the oscillation measurements^61^. As shown in Fig. 8A, the linear viscoelastic range was clearly shown in the amplitude sweep. In addition, the storage modulus (G′) was larger than the loss modulus (G″) under an electric field, indicating a higher degree of solid-like behaviour displayed by the ER fluid. When the strain amplitude was beyond a critical value, both G′ and G″ decreased sharply because of the breakdown of the structure of the ER fluid.

Figure 8B shows the G′ and G″ in the frequency sweep for the SiO_2_@Ni-Al LDH ER fluid in the linear viscoelastic region under different electric fields. The suspension exhibited solid-like behaviour, in which G′ was substantially larger than G″, and G′ remained almost constant over a broad frequency range. The larger G′ with increasing electric field strength reflects the higher ER effect^41^.

**Figure 9:**
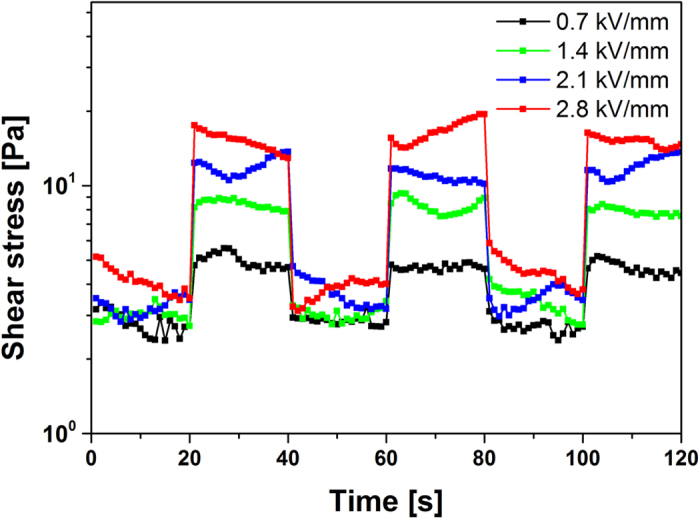
(**A**) Shear stress of SiO_2_/Ni-Al LDH composites based ER fluids at a fixed shear rate of 1 s^−1^ in the electric field with a square voltage pulse (t = 20 s).

In order to verify the sensitivity and the stability of the ER fluid to an electric field, the steady shear flow was operated at a fixed shear rate of 1 s^−1^ under applied square voltage pulse (t = 20 s). The shear stress under various electric field strengths is shown in Fig. 9. When exposed to electric field, the shear stress of the ER fluids jumped to higher level from the zero-field shear stress and dropped to zero-field level again right away when the electric field is removed. This phenomenon indicates the fast and reversible transition in the as-prepared ER fluid corresponding to the electric field.

## Methods

All chemical regents were used as received from commercial sources without further purification. Ultrapure water was made by the Flom ultrapure water system and used in all experimental and washing processes.

### Synthesis of SiO_2_ sphere and Ni-Al LDH sheets

The TEOS (≥28%, 4.37 g) was firstly added into ethanol (≥99.5%, 50 mL), then the mixture of 2.23 g H_2_O, 7.7 mL ammonium (28%) and 40 ml ethanol was dropwise added into the above solution. After 20 hours′ hydrolysis reaction, the mixture was separated in a high speed centrifugal machine to collect the precipitation. Then, the white SiO_2_ sphere was dispersed in ultrapure water and centrifuged to collect the white precipitate, this process was repeated until the supernatant became clear. The sample was further dried under high vacuum to obtain the pure SiO_2_ spheres. For the preparation of Ni-Al LDH sheet, certain amounts of Ni(NO_3_)_2_·6 H_2_O (99%, 1.308 g), Al(NO_3_)_3_·9 H_2_O (99%, 0.8622 g) and urea (99%, 0.9468 g) were added to 150 mL ultrapure water. Then the mixture was kept in an autoclave pressure vessel at 95 °C for 24 h. Finally, the solution were centrifuged and washed several times with ultrapure water. The clean Ni-Al LDH sheets were dried at 75 °C for 12 h in a vacuum oven. Before using, the Ni-Al LDH sheet dispersed in ethanol was broken using ultrasonic waves for 6 h.

### Preparation of SiO_2_@Ni-Al LDH composites by self-assembled process

SiO_2_@Ni-Al LDH composites were prepared by electrostatic adsorption between SiO_2_ sphere and Ni-Al LDH sheet. SiO_2_ sphere (0.1 g) was dispersed in 20 ml ethanol by ultrasonication for 15 min. The sodium dodecyl benzene sulfonate (SDBS) (98%, 0.01 g) was then added into the above solution, followed by magnetic stirring for 1 h. After centrifugation, the supernatant was discarded. The precipitation was dispersed in 20 ml ethanol under sonication. Dispersion of Ni-Al LDH sheet in ethanol (0.0044 g Ni-Al LDH sheet, 5 ml ethanol) was added into the above solution. The resulting mixture was stirred for 5 h and then centrifuged. The precipitate was collected and then dried at 60 °C for 12 h to afford SiO_2_@Ni-Al LDH composites.

### Characterization

Field-emission scanning electron microscopy (FE-SEM) (Model JSM-2010, JEOL) was used to observe the morphology of Ni-Al LDH sheet and SiO_2_@Ni-Al LDH composites. Transmission electron microscopy (TEM) (Model H-800, Hitachi, Tokyo, Japan) was performed to analyze the morphology of Ni-Al LDH sheets and SiO_2_@Ni-Al LDH composites. Scanning transmission electron microscopy (STEM) (Model JEM 2100 F, JEOL) was carried out to characterize the distribution of the elements. X-ray photoelectron spectroscopy (XPS) tests were measured using an ESCALAB MK-II electron spectrometer with an Al Kα X-ray source. X-ray diffraction (XRD) (Model DMax/rA, Rigaku, Japan) was performed using a rotating anode X-ray diffractometer with Cu Kα radiation (λ = 0.154 nm) at 40 kV. The ER characteristics were examined using a rotational rheometer (Physica MCR301, Anton Paar) equipped with a coaxial cylinder.

## Conclusions

The SiO_2_@Ni-Al LDH composites were successfully fabricated via self-assembly of Ni-Al LDH sheet on SiO_2_ sphere based on electrostatic interactions. SEM, TEM, EDX, STEM, XRD and XPS analyses were carried out to confirm the successful preparation of the core-shell SiO_2_@Ni-Al LDH composites. The SiO_2_@Ni-Al LDH composites displayed an enhanced ER characteristic compared to bare SiO_2_ suspension when dispersed in silicone oil, highlighting the potential applications of the SiO_2_@Ni–Al LDH composites in an automotive industry such as shock absorbers, engine mounts and clutches.

## Additional Information

**How to cite this article**: Ji, X. *et al*. Self-assembly preparation of SiO_2_@Ni-Al layered double hydroxide composites and their enhanced electrorheological characteristics. *Sci. Rep*. **5**, 18367; doi: 10.1038/srep18367 (2015).

## Figures and Tables

**Figure 1:**
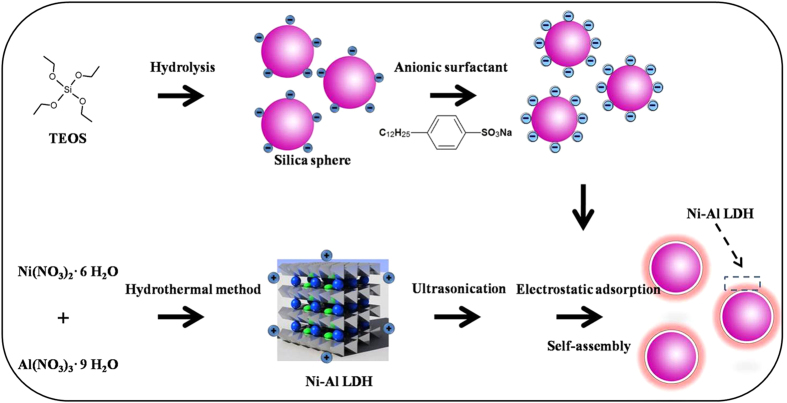
Schematic diagram of the stepwise preparation of electro-responsive SiO_2_@Ni-Al LDH composites.
